# Prediction of Turn-Ends Based on Anticipation of Upcoming Words

**DOI:** 10.3389/fpsyg.2012.00376

**Published:** 2012-10-01

**Authors:** Lilla Magyari, J. P. de Ruiter

**Affiliations:** ^1^Language and Cognition Department, Max Planck Institute for PsycholinguisticsNijmegen, Netherlands; ^2^Faculty of Linguistics and Literary Studies, University of BielefeldBielefeld, Germany

**Keywords:** conversation, turn-taking, timing, prediction, anticipation, gating, comprehension, production

## Abstract

During conversation listeners have to perform several tasks simultaneously. They have to comprehend their interlocutor’s turn, while also having to prepare their own next turn. Moreover, a careful analysis of the timing of natural conversation reveals that next speakers also time their turns very precisely. This is possible only if listeners can predict accurately when the speaker’s turn is going to end. But how are people able to predict when a turn-ends? We propose that people know *when* a turn-ends, because they know *how* it ends. We conducted a gating study to examine if better turn-end predictions coincide with more accurate anticipation of the last words of a turn. We used turns from an earlier button-press experiment where people had to press a button exactly when a turn-ended. We show that the proportion of correct guesses in our experiment is higher when a turn’s end was estimated better in time in the button-press experiment. When people were too late in their anticipation in the button-press experiment, they also anticipated more words in our gating study. We conclude that people made predictions in advance about the upcoming content of a turn and used this prediction to estimate the duration of the turn. We suggest an economical model of turn-end anticipation that is based on anticipation of words and syntactic frames in comprehension.

## Introduction

We use language most frequently in an informal, conversational setting. Despite this, most of the studies of language comprehension and production are based on experiments that are conducted in a laboratory with highly controlled language input, with single subjects. When one leaves the laboratory and takes a closer look at natural conversations, a striking feature emerges that has rarely been investigated experimentally. People are remarkably fast and accurate in switching between listener and speaker roles during conversations. In Dutch conversations, almost half of all turn-taking role transitions take place with a temporal offset of between −250 and +250 ms measured from the end of the current turn (de Ruiter et al., 2006). Such rapid turn-taking is not specific for Dutch conversations, but has been shown to be universal across cultures (Stivers et al., 2009). Yet, recent models of language comprehension and production (for example, Indefrey and Levelt, 2004; Hagoort, 2005; Pickering and Garrod, 2007) do not explain how the production/comprehension system manages to achieve this highly accurate timing.

Almost four decades ago, Sacks et al. (1974) suggested that it is a normative rule in conversations that participants respond as soon as the current speaker has finished. When there are departures from this rule, the gaps or overlaps are interpreted communicatively. For example, a short silence before a response can be a sign of disagreement in the coming response (Davidson, 1984; Pomerantz, 1984). They also argued that listeners must predict the end of the current turn to properly time their own turn. But from the point of view of the underlying cognitive processes, rapid turn-takings are puzzling. People are required to execute two major cognitive tasks during a conversation: they have to both comprehend one utterance and plan another. The short duration of turn-transitions suggest that comprehension and production must occur in parallel toward the turn-ends. Despite this complex process, every day conversations run smooth and effortlessly. The speed and sensitivity for timing of utterances makes the cognitive processes underlying conversations even more complicated. Next speakers also have to predict when a turn is going to end in order to time their own utterances correctly. How do people execute three major tasks in such a short time and how are people able to predict turn-ends with such accuracy?

Some proposals suggest that speakers produce *signals* that indicate that they are about to finish their turn (Duncan, 1974; Duncan and Fiske, 1977). Another account assumes that a potential next speaker can *anticipate* the moment when the current turn is going to end (Sacks et al., 1974). The “signaling” approach identifies cues that usually coincide with the end of turns, for example a certain intonation pattern, a drop in pitch or loudness at the end of phonemic clauses (Duncan, 1974). However, these turn-ending cues probably occur too late for the listener, who after all has to prepare a coherent answer as well. Experimental research on production of words and utterances shows that it requires at least 600 ms or more for the production system to arrive from the message level to articulation (Jescheniak et al., 2003; Indefrey and Levelt, 2004; Schnurr et al., 2006). Therefore, it appears very plausible that listeners must know more than a half a second in advance that a turn is going to end. While the signaling approach does not correspond to recent experimental results on language production, the anticipation account has also not provided a model for *how* turn-end projection is possible. It has been suggested, for example, that turn-ends can be anticipated in advance by a pitch peak that signals that the next syntactic completion point can be a turn-transition point (Schegloff, 1996). And a more recent experimental study (de Ruiter et al., 2006) has shown that the semantic and syntactic content plays a major role in turn-end predictions. But it has been not studied how the semantic-syntactic content helps in estimating the duration of turn-ends.

A few experimental studies of end-of-turn prediction concentrated mainly on the role of intonation versus the semantic and syntactic content of turns. Grosjean and Hirt (1996) used the gating method to investigate if people can predict when French and English sentences end. They presented sentences auditorily in segments of increasing duration while the subjects had to guess with how many words the fragments would continue in a multiple choice response task. The sentences were either short or they were expanded by optional noun-phrases. Participants could only predict whether the segments continued with three or six more words, when they heard the first potentially last word, i.e., at the first point in the sentence where the sentence could end if it was a short sentence without optional noun-phrases. Grosjean and Hirt concluded that prosodic information in English is made available for the prediction of sentence length but only when the semantic and syntactic information are of no help. This result shows that the semantic and the syntactic information play a role in turn-end anticipation. But the experiment used recordings of read sentences. As spontaneous speech differs from read speech (Levin et al., 1982; Esser and Andrzej, 1988), it is unclear to what degree their results can be generalized to account for processing of spontaneous speech.

de Ruiter et al. (2006) investigated the contribution of the lexical-syntactic content and intonation in turn-end predictions using recordings of natural conversations. They found that people rely on the lexical content and syntactic information in predicting turn-ends. Subjects listened to individual turns taken from Dutch telephone conversations and were asked to press a button exactly at the moment the turn-ended. The duration between the end of a turn and the button-press (called *bias*) was measured. In different experimental conditions, the turns were presented naturally (as recorded) or a modified version was played. In one of the conditions, the intonational contour was removed, in another condition the lexico-syntactic content was removed but the intonational information was left intact. When subjects were listening to the original turns, their button-presses coincided with the turn-ends accurately; the distribution of the button-presses was similar to the distribution of the duration of the turn-transitions in the original conversations. There was no change in accuracy when the intonational contour was removed, but the performance deteriorated significantly when the words could not be understood, even if the intonational information was still present in those stimuli. De Ruiter and his colleagues concluded that the intonational contour is neither necessary nor sufficient for the prediction of turn-ends. These results suggest that the symbolic (lexico-syntactic) information plays an important role in the prediction of turn-endings.

The results of this experiment correspond to the criticism that intonation cues seem to occur too late to be used for turn-end prediction. However, this does not mean that intonation is not used at all in turn-taking. It has been suggested that the pitch contour can also serve as a turn-keeping signal before a pause, indicating that despite the pause the turn has not finished yet (Caspers, 2003; de Ruiter et al., 2006). On the other hand, how exactly the lexical content and the syntax are helping in turn-end anticipation remains an open issue.

We propose that *anticipation* of the lexical content and the syntactic information helps in the prediction of the time when a turn-ends. In other words, people know *when* a turn-ends by predicting *how* it ends.

For a long time, predictions were not considered to be part of language processing because they were thought to be inefficient and cognitively demanding. But others have recently argued that predictions can help in speeding up comprehension and disambiguate the noisy linguistic input (Kutas et al., 2011). Experimental studies using eye-tracking and electrophysiological techniques revealed that predictions can be made at many linguistic levels during language processing. It has been shown that listeners can anticipate upcoming arguments of verbs (Altmann and Kamide, 1999; Kamide et al., 2003), the gender of words (Wicha et al., 2004; Van Berkum et al., 2005), and also the upcoming word forms (DeLong et al., 2005). Kutas et al. (2011) argue that electrophysiological studies show that word features and word forms get neurally preactivated in highly constraining contexts (DeLong et al., 2005).

**Figure 1:**
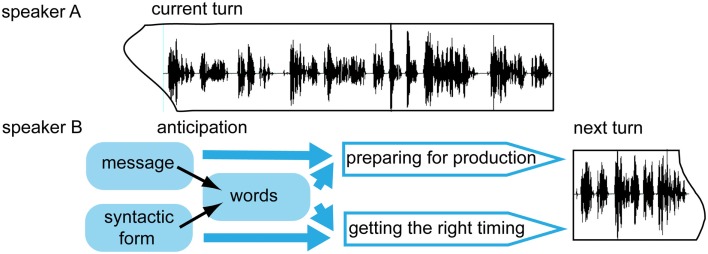
**Schematic model of prediction and production processes of the current listener/next speaker in conversations**.

Timed turn-transitions require prediction of turn-durations during every day conversation. We are interested if predictions of words and syntactic structures are also made during comprehension of everyday conversations and if these predictions help in predicting the duration of the conversational turns. In real life conversations, the context can also provide further information about the speaker’s intention (“message” of the turn) even before any prediction about words or syntactic frames are made. These different types of anticipated information could differentially influence the preparation of the production of next turns. When a message is anticipated, this may provide enough information to start the preparation of a response. When syntactic frames or words are anticipated, this could facilitate the accurate timing of the next turn’s production. Perhaps words can only be anticipated when both the message and the syntactic form are clear (Figure 1).

In order to test our hypothesis we conducted a gating experiment using the experimental stimuli of de Ruiter et al.’s (2006) study (these stimuli were single turns from recordings of natural conversations). We presented selected stimuli (single turns) from this study to participants, but cut them off at several points. The participants listened to the turn-fragments or to the entire turn and had to guess how the turn would continue. If they did not make a guess, they had to guess how many words would follow the fragment they heard in a multiple choice task. Our prediction was that accuracy of button-presses to a given turn-end in the earlier experiment correlates with the accuracy with which words the participants guessed follow the presented fragment. We test this prediction by using a mixed-effect model on the guessed words which are coded as binary responses (correct or not, PRED_END). We test if the accuracy of the button-press responses of the turns (BIAS) has an effect on the proportion of the correct guesses. It is possible that people can predict duration of turns not only by predicting the words coming, but also based on the syntactic structure even when no concrete word predictions are made. We assume that correct syntactic predictions correlate roughly to the expectations about how many number of words follow a fragment. For example, when certain syntactic elements require obligatory arguments, participants can predict that a certain number of words are still required for the turn being grammatically correct. Therefore, we predict that accuracy of button-presses correlates also with the predictions about how many words follow the fragments. We test this in two ways: (1) First, a mixed-effect model is used on the number of word predictions as binary responses (correct or incorrect number of words is predicted, NUM_CORRECT). It is tested if bias of turns (BIAS) has an effect on the proportion of the correct number of words prediction. (2) Then, another mixed-effect model is used on the number of word prediction as a continuous variable. Here, we code the difference in number of words between the predictions and the actual number of words to come (PRED_NUM). We test if BIAS has an effect on this difference. At the number of words predictions, we differentiate between the predicted number of words calculated from the entered text (free guesses) and the predicted number of words given in the multiple choice task. We run the analysis on both type of responses (once only on number of word predictions calculated from the entered text, and once on predictions based on the entered text and on multiple choice task together). In each of the analyses, the cut-off locations (CUT-OFF) are included as a dependent variable besides BIAS.

## Materials and Methods

### Participants

Fifty native speakers of Dutch (forty-two women and eight men, aged between eighteen and twenty-nine) participated in the experiment. The data from one subject was excluded because the results indicated that he did not understand the task correctly. The subjects were paid for their participation.

### Stimulus material

The experimental materials were selected from stimuli used by de Ruiter et al. (2006). In de Ruiter et al.’s study subjects listened to individual turns taken from Dutch telephone conversations and were asked to press a button exactly at the moment the turn-ended. We selected from those turns into our stimuli material. It was known for each turn from the results of the earlier study, how accurately subjects could predict the end of turns by button-press. We took this information into account in our selection. We used the value of *bias* at each turn that was calculated in the earlier study based on the subject’s responses. Bias is the temporal offset between the end of the turn and the button-presses. In de Ruiter et al.’s study, subjects did not react on the occurrence of a stimulus but subjects were trying to press the button exactly at the occurrence of the turn-end. This could result also in “early” responses that occur before turn-end. Therefore, subject’s button-press responses are called *bias* instead of “reaction time.” When bias is negative, the subject pressed the button before the end of the turn, when bias is positive the subject pressed the button after the turn. The averaged bias of a turn indicates how accurately subjects could *on average* predict the time when the turn-ended. A turn with a highly positive bias indicates that subjects pressed the button considerably after the turn-ended. A low bias (small positive value or with a small negative value) shows that subjects pressed the button on time or a little earlier than the turn-ended in average. We used this average bias calculated for each turn to select our stimuli.

**Figure 2:**
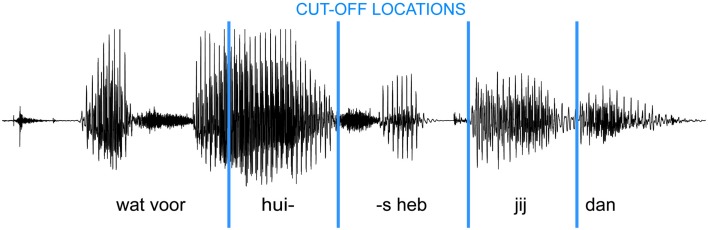
**Sound wave and content of an example turn with four cut-off locations**. The audio recording of each turn was cut at four different points (blue vertical lines) before the turn-end.

For the purposes of the present study, 20 turns with averaged biases ranging from low to high were selected from 10 different speakers (min = −18 ms, max = 330 ms, mean = 159 ms). It was observed in the earlier study that turns with longer duration tend to have a smaller (absolute) bias. In this study, we were interested if there is a relationship between bias and predictability of the last words of turns, therefore, we tried to avoid differences between turns with different bias caused by differences in the duration of the turns, in the duration of the fragments of turns to be predicted, in the number of words, and in the number of syllables to be predicted. Therefore, we selected turns with the following procedure: Initially, we selected pairs of turns having approximately the same duration, but with one turn with high and one with lower bias. For each turn, four versions were made by cutting off the speech at four different temporal locations (Figure 2).

The cut-off locations within each pair were at the same points in time measured from the end of the recordings, but they were different across stimuli pairs. Locations of the cut-off points were determined in each pair according to the boundaries of the two last words of each of the pairs. The turn-pairs with approximately similar duration but with different bias ensured that the bias of the selected turns did not correlate with their duration (*r* = −0.05, *p* = 0.82). The pairing of turns were not used further in the analysis of the results because we used bias as a continuous variable but the cut-off procedure based on the pairs ensured that there was no systematic (linear) relation between the bias of the turns and other features of the fragments that had to be predicted. At each cut-off location (from the longest fragment to the shortest across turns), there was no correlation between the bias of the turns and the duration of the cut-off fragments (cut-off 1: *r* = 0.55, *p* = 0.14, cut-off 2: *r* = 0.03, *p* = 0.89, cut-off 3: *r* = −0.12, *p* = 0.63, cut-off 4: *r* = −0.32, *p* = 0.22), the number of words to be predicted^1^ (cut-off 1: *r* = −0.06, *p* = 0.81, cut-off 2: *r* = 0.03, *p* = 0.88, cut-off 3: *r* = −0.07, *p* = 0.76, cut-off 4: *r* = −0.1, *p* = 0.71), the number of syllables to be predicted^2^ (cut-off 1: *r* = 0.04, *p* = 0.85, cut-off 2: *r* = 0.06, *p* = 0.79, cut-off 3: *r* = −0.04, *p* = 0.86, cut-off 4: *r* = −0.01, *p* = 0.97), whether the cut-offs were at word-boundaries or not (cut-off 1: *r* = −0.17, *p* = 0.48, cut-off 2: *r* = 0.08, *p* = 0.75, cut-off 3: *r* = −0.18, *p* = 0.44, cut-off 4: *r* = −0.1, *p* = 0.71), and average frequency of turns to be predicted (cut-off 1: *r* = −0.01, *p* = 0.95, cut-off 2: *r* = 0.39, *p* = 0.1, cut-off 3: *r* = 0.19, *p* = 0.46, cut-off 4: *r* = 0.34, *p* = 0.21). Word frequency was based on the log lemma frequencies of the CELEX database (webcelex) of the Max Planck Institute for Psycholinguistics. The frequency of two words which were not found in CELEX was set to 0. As a result of the cut-off procedure each turn had four versions with increasing cut-off fragments from the end, but the exact duration of the cut-off fragments at each location varied across turns. Table 1 shows the minimum, maximum, and average duration of the cut-off fragments (i.e., the duration from the cut-off location to the end of the turn) at all four locations. The duration of the entire (non-cut) turns varied between 1.06 and 2.04 s with mean of 1.48 s.

**Table 1:** **Duration, number of words, and number of syllables of the cut-off fragments across turns**.

Cut-off	Duration (cut-off from end; in ms)			Number of words to predict			Number of syllables to predict		
	Min	Max	Mean	Min	Max	Mean	Min	Max	Mean
Fourth (shortest)	73	355	195	0.5	1	0.794	0	2	0.882
Third	146	641	343	0.5	3	1.2	0	4	1.7
Second	217	821	470	1	3.5	1.8	1	5	2.4
First (longest)	361	1237	711	2	5	2.875	2	9	4

Table 2 shows two turns and their English translation with the locations of the cut-offs. Vertical lines indicate where the recordings were cut in the different versions. Notice, that for each turn, two of the cut-off locations were (1) at the word before the last word and (2) before the last word.

**Table 2:** **Two examples of the experimental stimuli with cut-off locations**.

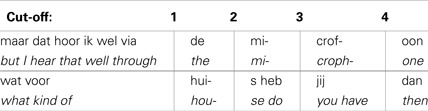

### Experimental design

Subjects were randomly assigned to one of five experimental lists. The stimuli in the lists were presented in random order to each subject. Their task was to indicate (on a computer terminal) if the presented segment constituted a complete turn. If the subjects decided that the turn was not complete, they were asked to guess and enter the text they believed would complete the turn. If they were unable to guess how the turn continued then they were presented with a multiple choice task. They were asked to guess with how many words the turn would continue. The subjects were allowed to choose between the following options; (A) one word, (B) two words, or (C) three or more words. Subjects were also asked how certain they were of their responses. They had to indicate this on a four-point likert scale.

### Procedure

Subjects were seated in front of a computer screen and a keyboard with headphones. The instructions were visually presented on the screen. Before each stimulus a sentence was presented on the screen in Dutch, saying: “When you press the space bar you can listen to the next sound fragment two times.” Five hundred milliseconds after pressing the space bar, a stimulus was presented two times, with a 1500 ms pause between the two presentations. After the stimulus presentation, the subjects were shown a prompt (>:) on the screen where they were required to type their guess about the continuation of the fragment. If they thought the turn that they were listening to was complete, they had to type: “.”. If they were unable to guess how the turn continued, but they did not think that the turn had finished, they were asked to type a “-”. When they were unable to guess about the continuation, they were presented with the multiple choice task of number of words predictions. After reading the instructions, the participants did a training session. During the training four stimuli were presented which were not parts of the experimental lists. After the training session, which could include verbal clarifications, the experimenter left the room and the participants could continue the experiment alone.

### Data-coding

Two variables, PRED_END and PRED_NUM were created based on the responses entered. The variable PRED_END (prediction of the rest of the turn) was set to 1 when the continuation of the turn was entirely correct. We regarded a response entirely correct when the guess exactly matched the continuations. Usage of synonyms or words from the same syntactic category did not count as a correct response. It was also set to 1 when it was indicated correctly that the turn has ended. PRED_END was set to 0 when the typed-in continuation was incorrect (different words, or more, or less words were guessed) or participants were unable to provide a guess.

The PRED_NUM (prediction of the number of words) variable represented the difference between the number of guessed words and the number of words actually completing the turn. This value was calculated from the number of words that were entered or if no guess was provided, from the estimation of how many words would complete the turn in the multiple choice task. In this task, the maximum number of words that could be chosen was “three or more words.” Therefore, when the difference was more than plus or minus three words, the value of PRED_NUM remained plus or minus three. So, the values of this variable ranged between −3 and 3. When the exact difference in the number of words could not be clearly identified (for example, when two words remained to complete the turn, and the participant chose the option “more than three words”), a value with the smallest possible difference was given (in this case, +1).

### Statistical analysis

Some responses (less than 1% of all data) that were not clear (e.g., words that do not exist in the Dutch language were typed in) were excluded from the analysis. It was assumed that recognition that a turn-ended and prediction of words that continue a fragment are different types of tasks. Therefore, the responses given at the full turns were analyzed separately from the responses given at fragments of turns. It was also checked whether the proportion of answers for the different analyses were significantly different from each other using a proportion test.

The results were analyzed using a Generalized Linear Mixed Model (GLMM) for the binary response variable (PRED_END) and a Linear Mixed Model (LMM) for continuous response variable (PRED_NUM) with Restricted Maximum Likelihood (Baayen, 2008; Jaeger, 2008). The analysis was performed with the lmer function of the lme4 package (Bates and Maechler, 2009) in R Development Core Team (2009). For the GLMM, binomial error structure and a logit link function was specified. At each model, we included BIAS, CUT-OFF, and their two-way interaction as fixed variables. The variable BIAS contained the average temporal offset between a turn-end and the button-presses from de Ruiter et al.’s study, CUT-OFF was an index of a cut-off location in a turn ranging from 1 (longest cut-off) until 4 (shortest cut-off, closest to turn-end). Initially, GENDER, AGE, and ORDER (order in which trials were presented) were included as fixed variables that could confound the results. We included SUBJECT (subject’s ID) as random effect. The model simplification worked in the following way: When the three confound variables were not significant, they were removed from the model. Then, when the two-way interaction was also not significant it was also removed. Once a final model was reached, the model was computed again with the lmer function but using Maximum Likelihood and compared to a null model comprising only the random effect. For the model comparison, the R-function ANOVA (Crawley, 2007) that applies a chi-square test was used. When the final model was significantly different from a null model, the *p*-values of the estimates were examined. For GLMM, *p*-values of the coefficients were derived from the *p*-values provided by the output of the lmer function. For LMM, *p*-values were derived using pvals.fnc R-function that estimates *p*-values based on Markov chain Monte Carlo sampling (with 1000 samples). Significant interaction effects were further analyzed with using a linear regression model. The model was also evaluated with the ANOVA function that shows by an *F*-test if the independent variable contributes significantly in explaining the variance in the dependent variable (Baayen, 2008).

## Results

### Recognition of turn-ends

In 92% of the cases (*n* = 196), it was correctly recognized that a turn-ended. In the GLMM, PRED_END (correct or not correct recognition of a turn-end) was a binary response variable. CUT-OFF as a fixed factor was not included because only responses at turn-ends were analyzed. In the final model, BIAS was included as a fixed effect and SUBJECT as a random effect. This model (including BIAS and SUBJECT) was significantly different from a model containing only the random effect [SUBJECT; chi-square(1) = 4.66, *p* = 0.03]. The estimate of the coefficient of BIAS shows that turn-ends were recognized better when the turns had a higher bias, but this effect did not reach significance (β = 0.01, *z* = 1.86, *p* = 0.06). Therefore, it is possible that there is no difference in the recognition of turns end among turns with different bias.

### Prediction of the continuations

**Figure 3:**
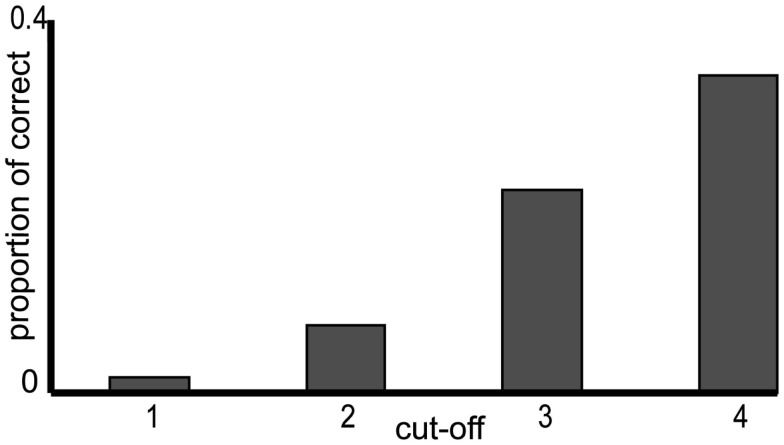
**Proportion of correct answers at each cut-offs**. As the index of cut-off increases so it is closer to the turn-end. When the cut-off is closer to turn-end, the proportion of correct answers increases.

**Figure 4:**
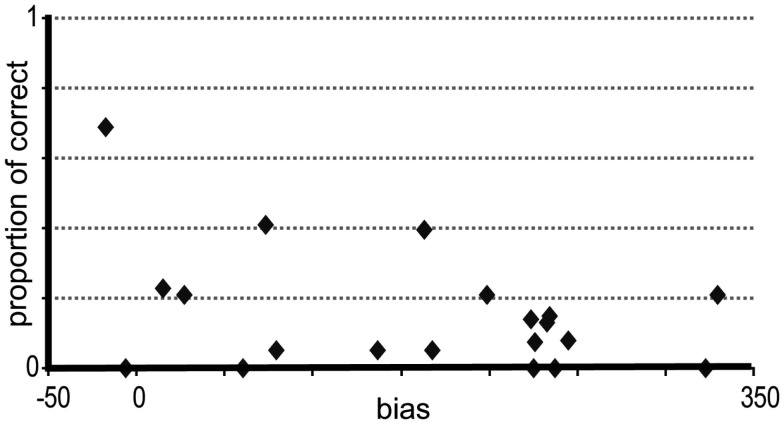
**Proportion of correct answers at each turn**. The *y*-axis shows the proportion of correct answers across cut-off locations. The *x*-axis shows the bias of each turn. When a turn has a larger bias, the proportion of the correct answers is lower.

Responses for fragments and not for full turns were analyzed. We excluded also three fragments that ended so close to the end of the turn that there was no audible information to be guessed. In the final model, BIAS and CUT-OFF were included as fixed effects [chi-square(1) = 87.46, *p* < 0.001]. A model including the two-way interaction between BIAS and CUT-OFF was not significantly different from the model without this interaction [chi-square(1) = 0.54, *p* = 0.46]. BIAS and CUT-OFF had an influence on the proportion of correct responses. When the CUT-OFF (β = 1, *z* = 8.22, *p* < 0.001) location was closer to the turn-end, the proportion of the correct answers increased. Figure 3 shows the proportion of correct answers at each cut-off location. When BIAS (β = −0.01, *z* = −4.8, *p* < 0.001) was higher, the proportion of correct answers decreased. Figure 4 shows the proportion of the correct answers for each turn.

### Prediction of the number of words

**Figure 5:**
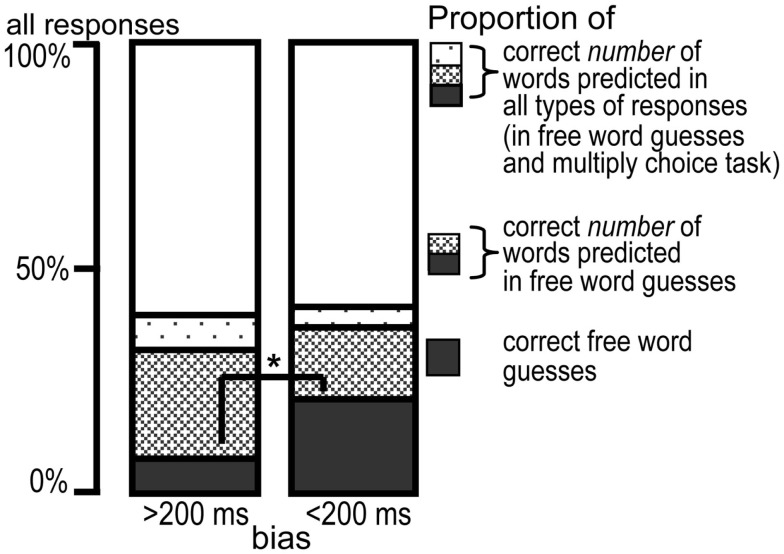
**Proportion of entirely correct guesses (dark bars), correct number of words prediction in the free word guesses (dark bars and diagonally striped bars), and correct number of words predictions in both types of responses, in free word guesses and in the multiple choice task responses (dark, diagonally striped, and dotted bars together)**. The proportions are shown for turns with lower bias (right bars) and higher bias (left bars). The two groups of turns were created only for illustration. The * shows the significant effect of bias only at the proportion of entirely correct continuations with free word guesses (dark bars). There was no significant effect of bias on the proportion of the correct number of words.

Our question was also if BIAS predicts the correct number of words. We excluded again those items where the full turn was heard. First, we analyzed the number of the predicted words in the free word guesses (in contrast to guesses where they did not type in words but guessed the number of words). The proportion of guesses with correct number of words was significantly larger than the proportion of entirely correct guesses [words = 15%, number of words = 35%, chi-squared(1) = 78.51, *p* < 0.001]. We created a new binary responses variable, NUM_CORRECT. When the number of words was correctly predicted, NUM_CORRECT was set to 1, when the number of words was incorrect or no words were entered it was set to 0. We fitted a GLMM using the procedure described in section “Statistical Analysis”. None of the confound variables and the interactions showed a significant effect, so the final model contained BIAS and CUT-OFF as fixed effects and SUBJECT as random effect [chi-square(2) = 53.64, *p* < 0.001]. Figure 5 shows the proportion of correct number predictions calculated from the entered text guess for turn with higher and lower bias. The figure compares the proportion of correct text guesses in terms of number of words collapsing completely correct guesses (solid dark bars) and correct number of words entered as text (solid dark bars and bars with diagonally striped pattern together). The GLMM showed that the proportion of correct number of words increased (β = 0.53, *z* = 7.09, *p* < 0.001), when the index of CUT-OFF became larger. To sum up, this analysis showed that lower bias did not correlate with predicting the number of words better in the free guesses. But the proportion of correct number of words predictions got higher toward the turn-ends.

We also examined if including the number of words estimations in the multiple choice task would change the effect. It was important to examine this because the proportion of all responses where the number of words was correct was significantly different from correct number of word predictions calculated from the entered text responses [words = 35%, all = 41%, chi-squared(1) = 5.26, *p* = 0.02]. In this analysis, NUM_CORRECT was set to 1 when the number of words was correct based either on the entered text or on the multiple choice number task, and it was 0 when the number of words were not correct. The final model contained BIAS and CUT-OFF [chi-square(2) = 22.81, *p* < 0.001] as main effects. A model with their interaction was not different [chi-square(1) = 0.58, *p* = 0.44]. The effect of BIAS was not significant (β = 0, *z* = 1.49, *p* = 0.14; Figure 5, dark, stripped, and dotted bars together), but CUT-OFF had an effect (β = 0.32, *z* = 4.59, *p* < 0.001). When index of CUT-OFF increased, also the proportion of correct number of words (in free guesses and in the multiple choice task together) increased.

**Figure 6:**
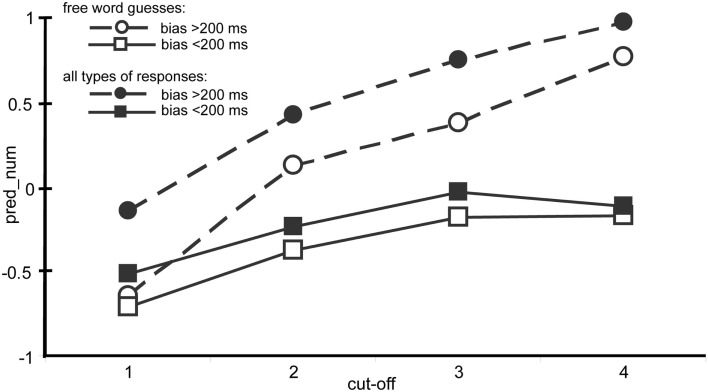
**Average of the difference between number of words predicted and continued a turn segment (PRED_NUM) at each cut-off at turn with lower (squares) and with higher (circles) bias among free word guesses (open squares and circles) and among both types of responses, free word guesses and multiple choice task responses (full squares and circles)**. Both of the analyses (free word guesses and all types of responses) show that the difference in PRED_NUM is increasing between the turns with lower and higher bias toward the turn-end. The two groups of turns were created only for illustrating the effect in the figure.

We also examined if there was a linear relation between the number of predicted words and BIAS. First, we inspected the number of word predictions among entered text guesses. These were 74% percent of all responses. PRED_NUM indicated the difference between the number of words predicted and the number of words in the continuation of turns. When PRED_NUM was negative, lower number of words was predicted than the number of words in the continuations. In the final LM model, BIAS and CUT-OFF and their interaction were included [chi-square(3) = 75.18, *p* < 0.001]. The interaction effect was significant (*t* = 3.58, *p* < 0.001), but not the main effects [BIAS: *t* = −1.27, *p* = 0.21, CUT-OFF (*t* = 1.16, *p* = 0.25)]. To examine the interaction, a linear regression model was fitted at each cut-offs. BIAS did not have a significant effect at the first cut-off locations [*F*(1) = 0.29, *p* = 0.59], but it had an effect at all the other cut-offs [2: *F*(1) = 10.13, *p* = 0.002, 3: *F*(1) = 10.03, *p* = 0.002, 4: *F*(1) = 31.27, *p* > 0.001]. At these cut-off locations, when BIAS was higher, the number of predicted words also became higher (2: β = 0.003, *t* = 3.18, *p* = 0.002, 3: β = 0.003, *t* = 3.17, *p* = 0.002, 4: β = 0.004, *t* = 5.59, *p* < 0.001). Figure 6 shows the average difference in number of words predictions at each cut-off locations between turns with higher and lower bias. The relevant data are the comparison of the open squares (turns with lower bias) and the open circles (turns with higher bias) in the figure. It shows that at the first cut-off point, there is hardly any difference between turns with different bias, but a difference emerges at later cut-off points. [Please, note that the grouping (turns with higher versus lower bias) is done only for visualization in the figure, the statistics was done on BIAS as a continuous variable].

When predictions of the number of words among all responses (containing either entered text or the multiple choice task) were investigated the results showed the same tendency as the earlier analysis. The final LM model contained PRED_NUM as response variable, BIAS and CUT-OFF and their interaction as fixed effects, and SUBJECT as random effect [chi-square(3) = 92.8, *p* < 0.001]. The main effects of BIAS (β = 0, *t* = 0.31, *p* = 0.76) and CUT-OFF (β = 0.06, *t* = 0.84, *p* = 0.41) were not significant but their interaction (β = 0.001, *t* = 3.04, *p* = 0.002) was significant. The linear regression model did not show an effect of BIAS at the first cut-offs [*F*(1) = 1.68, *p* = 0.2], but it showed an effect at all the other locations [2: *F*(1) = 12.19, *p* < 0.001, 3: *F*(1) = 25.69, *p* < 0.001, 4: *F*(1) = 40, *p* < 0.001]. Figure 6 shows the relevant data in the comparison of the full circles (turns with higher bias) and full squares (turns with lower bias). It shows that the difference between turns is getting larger with lower and higher bias at cut-off locations closer to turn-end. The mean of the number of words predicted (PRED_NUM) was larger than 0 at turns with bias higher than 200 ms [*t*(319) = 6.05, *p* < 0.001, mean = 0.45], and it was less than 0 at turns with bias lower than 200 ms [*t*(427) = −4.18, *p* < 0.001, mean = −0.22]. This means that when more words were predicted (compared to how many words is to come) the duration of the turn was estimated to be longer than its real duration. When fewer words were predicted, the duration of the turns were estimated closer to the actual end of the turn by the button-presses.

## Discussion

We examined whether people know when a turn-ends because they know how it ends. Therefore, we used a gating method to study how well people can predict the continuation of turn-fragments. Based on an earlier button-press experiment, it was already known how accurately the duration of those turns can be estimated.

The results show that the proportion of correct guesses about the not-heard words increased as the cut-off approached turn-ends. The proportion of correct guesses was also higher when a turn-end could be estimated better in time (i.e., it had a lower bias in the earlier button-press experiment). A linear relationship was also found between the bias in the earlier experiment and the difference in number of words people predicted compared to the number of words the fragments continued with. When bias was higher, more words were predicted, when bias was lower, fewer words were predicted.

The results suggest that people make predictions in advance about which words and how many words will follow a partially heard turn, and that they use this prediction in estimating the remaining duration of that turn. Importantly, we show that natural language use is predictable to a certain degree, and we suggest that such predictions are crucial for timed social, verbal interactions. Altogether, the proportion of the correct guesses is not high. However, our criterion for a correct guess was strictly the exact match between the predicted and the coming words. No synonyms or words from the same category were regarded as correct. Moreover, an off-line study perhaps only partially reflects the on-line prediction processes. The turns were also presented without their conversational context. The results also suggest that people follow their prediction in estimation of turn-duration even when those predictions are not entirely correct. This effect has been shown already at the second location of cut-offs that were around 340 ms before turn-ends on average (see Table 1).

One challenge of the anticipatory comprehension account is the explanation of what happens when mispredictions are made. In order to avoid major misunderstandings there must be a monitoring process that compares the actual input to the predicted input. This is compatible with studies from Kolk et al. (2003) who suggest that the input is monitored by the language perception system (Van de Meerendonk et al., 2011). Interestingly, our results show a correlation between button-presses and the anticipated information. If the actual input is continuously monitored, how is it possible to predict turn-ends based on wrongly anticipated information? And if people follow what they wrongly anticipate, why do they not end up with continuous misunderstanding? We can only speculate on possible answers for these questions, but further work could give more insight.

When people predicted more words than the number of words that were actually in the turn, button-presses were also late. Late button-presses could have been caused by waiting too long and executing the movement too late, only after noticing the lack of continuation. Late responses also give time for re-planning the production in a conversational situation. In this case, mispredictions may lead to late answers but they do not necessarily lead to misunderstanding or non-relevant responses.

In our data, turns with lower bias (between −18 and 200 ms) were associated with “lower number of words” predictions. The button-press results can reflect that when people are predicting fewer words, they prepare for the movement earlier than necessary. A monitoring process can help to stop the movement execution and delay the response until the appropriate moment. Response preparation leads also to faster reaction times (Niemi and Näätänen, 1981). But in other cases, when the language perception system shows that unexpectedly the turn is not ending, it might still be difficult to stop the movement. It has been showed that there is a temporal boundary, a “point-of-no-return” in response preparation and execution (Logan and Cowan, 1984; Sosnik et al., 2007). Ladefoged et al. (1973) showed that it is also difficult to interrupt one’s own speech especially while a person is planning articulation. This could also explain why non-intentional overlaps could occur in conversations. In these cases, early responses may begin with non-relevant or incorrect responses (false starts) but they do not necessarily lead to misunderstanding. It is possible that the speaker has already noticed the misprediction and corrected but could not stop the articulation process. In our stimuli, we did not include turns with very early bias (<−200 ms), however, those occurred also in de Ruiter et al.’s (2006) experiment.

Using intonational cues may seem to be a simpler mechanism for predicting turn-ends but the account presented here is economical in terms of cognitive processing load. It explains how people are able to perform many simultaneous tasks before they start their turn. A next speaker in a conversation has to both comprehend the current turn and formulate and time their own subsequent utterance appropriately. Response preparation takes time and therefore it has to start before the previous turn-ends in order to avoid gaps. Response preparation, however, can be initiated only if the speaker knows roughly how to respond. Therefore, the next speakers have to anticipate not only when a turn-ends but also the content of the turn. When the last words of a turn can be anticipated, this provides information about both the content and the duration of the turn. Therefore, anticipatory comprehension is the very same process that helps to formulate the next turn and also to time it properly. This predictive mechanism provides a single, economic solution for the three major cognitive tasks that a listener needs to perform more or less simultaneously: (a) comprehending a turn, (b) preparing to produce the next one, and (c) estimating the end of the current turn.

Our data also shows that predictions are made not only about word forms but also about the number of words. We believe that the ability of our participants to predict the number of words reflects their ability to perform syntactic predictions that can help also in estimating turn-durations (see Figure 1 in the Introduction). Timing of turns is also probably influenced by other factors independent of anticipation. Overlaps and delays could also occur with communicative intent and gaps can also occur due to delays in the production or comprehension process of the speaker. But the timing of turns is frequently highly accurate in real life conversations. We suggest that this is possible only when people predict the syntactic form and word forms of turns before the turns end. It is an interesting issue for further research how duration of predicted linguistic elements is represented, for example, how precise duration estimations are and how much these estimations can be influenced by contextual information, for example, by the speaker’s speech-rate.

In our experiment, we showed that people were even able to guess upcoming words and the number of words of turns that were taken out of their conversational context. Button-presses for such “out-of-context” turns were also accurate. In real conversation, the context can facilitate anticipation even further. Listeners not only need to, but are also able to predict the continuation of the speaker’s turn before they are completed, and this ability is necessary for engaging in fluent verbal interactions.

## Conflict of Interest Statement

The authors declare that the research was conducted in the absence of any commercial or financial relationships that could be construed as a potential conflict of interest.

## Footnotes

^1^We computed the number of words to be predicted at each fragments of each turn. It was 1 when one full word had to be predicted, 2 when two words and so on. When the cut-off happened in a word, a half point was given for that word. For example, when the fragment was cut in the word before the last one, the value of WORDS was 1.5.

^2^We computed the number of syllables to be predicted at each fragment of each turn. It was 1 when one full syllable had to be predicted, 2 when two syllables had to be predicted and so on. We did not count a syllable to be predicted, when the vowel of the syllable could be already heard in the recording.
